# The Ability to Regulate Transmembrane Potassium Transport in Root Is Critical for Drought Tolerance in Barley

**DOI:** 10.3390/ijms20174111

**Published:** 2019-08-22

**Authors:** Kangfeng Cai, Huaizhou Gao, Xiaojian Wu, Shuo Zhang, Zhigang Han, Xiaohui Chen, Guoping Zhang, Fanrong Zeng

**Affiliations:** 1 Institute of Crop Science, Zhejiang University, Hangzhou 310058, China; 2 Zhejiang Academy of Agricultural Sciences, Hangzhou 310021, China

**Keywords:** drought tolerance, ion flux, transmembrane K^+^ transport, plasma membrane H^+^-ATPase, barley

## Abstract

In this work, the effect of drought on K^+^ uptake in root and its translocation from root to shoot was investigated using six barley genotypes contrasting in drought tolerance. Results showed that drought conditions caused significant changes in K^+^ uptake and translocation in a time- and genotype-specific manner, which consequently resulted in a significant difference in tissue K^+^ contents and drought tolerance levels between the contrasting barley genotypes. The role of K^+^ transporters and channels and plasma membrane (PM) H^+^-ATPase in barley’s adaptive response to drought stress was further investigated at the transcript level. The expression of genes conferring K^+^ uptake (*HvHAK1*, *HvHAK5*, *HvKUP1*, *HvKUP2* and *HvAKT1*) and xylem loading (*HvSKOR*) in roots were all affected by drought stress in a time- and genotype-specific manner, indicating that the regulation of these K^+^ transporters and channels is critical for root K^+^ uptake and root to shoot K^+^ translocation in barley under drought stress. Furthermore, the barley genotypes showed a strong correlation between H^+^ efflux and K^+^ influx under drought stress, which was further confirmed by the significant up-regulation of *HvHA1* and *HvHA2*. These results suggested an important role of plasma membrane H^+^-ATPase activity and/or expression in regulating the activity of K^+^ transporters and channels under drought stress. Taken together, it may be concluded that the genotypic difference in drought stress tolerance in barley is conferred by the difference in the ability to regulate K^+^ transporters and channels in root epidermis and stele.

## 1. Introduction

Drought is the most adverse environmental constraint that severely affects crop productivity worldwide [1]. As a result of differences in its severity and duration, drought causes an adverse impact on crop yield to different degrees, which may exceed all the other abiotic stresses [2]. It has been predicted that the worldwide drought frequency will likely increase more in presently dry regions by the end of the 21^st^ century due to the ever-changing climate [3], which tends to worsen the situation. Additionally, world population is expected to increase by one billion by 2030 and over 2.2 billion by 2050 [4]. Global crop production must be increased by 70 percent by 2050 to feed the world population [5]. Therefore, there is an imperative and urgent need to stabilize agricultural production and ensure food security. To achieve this target, exploring mechanisms underlying crop drought tolerance is the first step.

Drought primarily disturbs water uptake and aggravates dehydration of the crop by disrupting the cellular osmotic equilibrium. This gradually develops into various physiological and metabolic disorders such as damaged photosynthetic activity and induced oxidative stress, all of which consequently result in yield reduction [2]. Considerable evidence has proven that genetic variability of sensitivity to drought conditions exists between or within crop species [6,7]. Thus, it is promising to use the contrasting species or genotypes to discover the morphological, physiological and biochemical features that are consistently associated with drought tolerance [8,9,10]. Barley (*Hordeum vulgare* L.) is the fourth largest cereal crop worldwide with multipurpose use as animal feed, human food and brewing material. In addition, barley is ideal for genetic studies because of its simple genetic background [11] and known reference genome [12]. Furthermore, there exists a wide ecological range of barley which differs in water availability, temperature, soil type, altitude and vegetation, generating a high potential for adaptive genetic diversity against abiotic stresses, such as salinity and drought [13]. Recently, great effort has been made in investigating the drought tolerance mechanisms in barley and as well as breeding drought-tolerant cultivars [14,15]. However, domestication of this crop has imposed a marked truncation on the genetic variation present in wild populations, as only 40% of the alleles found in wild barley are present in the cultivars [15]. Therefore, as an untapped genetic reserve, the wild-barley germplasm provides promising genetic variation for crop improvement.

Plants have developed a series of sophisticated and effective strategies to adapt to drought conditions. Among these, osmotic adjustment coupled with induction of abscisic acid and dehydrins have conferred drought tolerance of plants by maintaining the high water potential in tissues [16]. Osmotic adjustment can be achieved by synthesizing low weight organic osmotic compounds, such as soluble sugars, amino acids, methylated quaternary ammonium compounds, polyamines, polyols and cyclitols [17]. Numerous studies have investigated the important roles of these low weight organic osmotic compounds in improving plant tolerance to drought conditions. Exogenous application of glycine betaine improved growth upon drought treatment in *Phaseolus vulgaris* [18], *Glycine max* [19] and *Zea mays* [20]. Garg et al. [21] have provided evidence for successful conferment of drought tolerance to rice by means of overexpression of trehalose biosynthetic genes. However, the synthesis of organic osmotic compounds is generally a slow process costing a massive amount of energy and assimilation substance, and is likely to function only when severe dehydration occurs, suggesting that this kind of osmotic adjustment may be critical for plant survival, rather than the improvement of plant growth and crop yield under drought stress [17,22]. 

Compared with energy- and assimilate-consuming synthesis and accumulation of organic osmotic adjustment substances, taking up inorganic ions from the environment to increase cytosolic ion concentrations through transporters and channels is considered as a much more cost-effective way for intracellular osmotic adjustment [23]. Potassium (K^+^) is the most abundant cation in plants and comprises 2% to 10% of a plant’s dry weight [24]. It is not only an essential element for the physiological and biochemical processes of plants such as enzyme activation, protein biosynthesis, photosynthesis, osmotic potential regulation, valence neutralization, pH stabilization and stomatal movement, but is also involved in plants’ adaptive response to abiotic or biotic stresses, including drought, salinity, cold, frost, waterlogging, diseases and herbivore [25,26,27]. Potassium fertilization has been shown to alleviate the adverse impacts of drought stress in rice [28], wheat [29], faba beans [30] and cotton [31]. Recently, Hosseini et al. [32] found that besides being associated with leaf water status, potassium is also associated with the concentration of starch and other primary carbon metabolites, as well as being involved in ABA homeostasis and carbohydrate metabolism under drought stress. All these results suggest that potassium plays a critical role in plants’ adaptive response to drought stress. It has been demonstrated that a larger amount of K^+^ uptake conferred a higher level of drought tolerance in *Arabidopsis* [33] and barley [34]. However, many questions remain unanswered regarding the mechanism in potassium homeostasis in regulating drought tolerance in barley; for example, what is the primordial responses of net ion fluxes to drought stress? Is the ability of K^+^ transmembrane transport conferring the genotypic difference in drought tolerance in barley? If yes, what kind of K^+^ transporters or channels are involved in the drought tolerance?

The aim of this study is to fill the above gaps in our knowledge. To achieve this, we used contrasting cultivated and wild barley genotypes to investigate the genotype-specific effect of drought on K^+^ uptake in root and translocation from root to shoot. Also, the role of K^+^ transporters and channels and plasma membrane (PM) proton pumps in the adaptive response of barley to drought stress was investigated by gene expression quantification. Our results showed that drought conditions caused a significant uptake of K^+^ in a time- and genotype-specific manner, affecting K^+^ regulation. This led to the conclusion that the genotypic difference in drought stress tolerance in barley is conferred by the difference in the ability to regulate K transporters and channels in root epidermis and stele.

## 2. Results

### 2.1. Barley Genotypes Differ Significantly in Their Sensitivity to Drought

**Figure 1:**
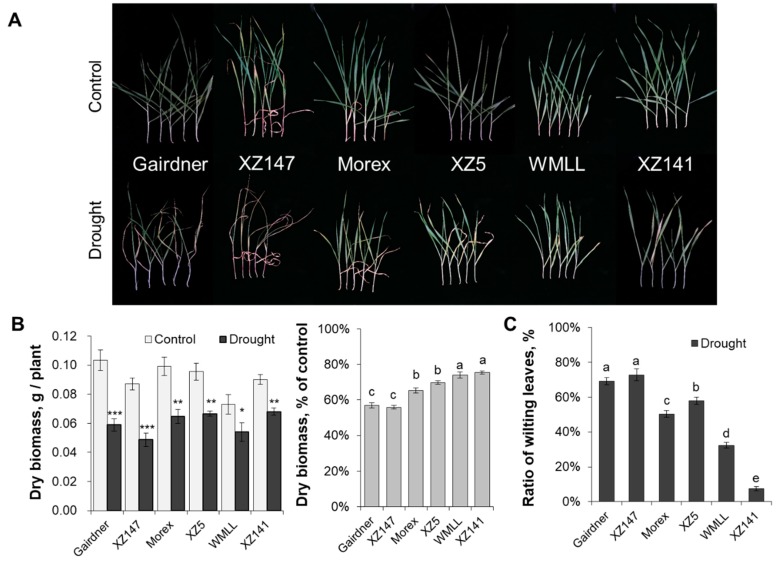
Effect of water-limited drought stress on plant growth of barley seedlings. Twelve-day-old barley seedlings were subjected to drought stress. Plants were harvested for morphological (**A**), biomass (**B**) and wilting leaves (**C**) analysis at 10 d after onset of treatments. Vertical bars represent means ± SE of five replicates. Asterisks indicate the significant difference between treatments at * *p* < 0.05, ** *p* < 0.01 and *** *p* < 0.001. Different lowercase letters indicate the significant difference between genotypes at *p* < 0.01.

A whole-plant experiment was undertaken to assess the genotypic variation in a range of barley genotypes under drought stress. Ten days of drought stress affected the growth of all barley genotypes, but to different extents. The most severe effect of drought on the plant growth was observed in genotypes Gairdner and XZ147, followed by Morex and XZ5, whereas WMLL and XZ141 suffered the least severe impact (Figure 1A). These genotypes were therefore termed drought sensitive (Gairdner and XZ147), moderate tolerant (Morex and XZ5) and tolerant (WMLL and XZ141). The average shoot dry weights of tolerant, moderate and sensitive genotypes were reduced by 25%, 33% and 44% relative to the control (Figure 1B) respectively. A significant wilting sign was also induced by drought stress. The ratios of wilting leaves were 20%, 54%, and 71% on average under drought treatment for tolerant, moderate, and sensitive genotypes, respectively (Figure 1C).

**Figure 2:**
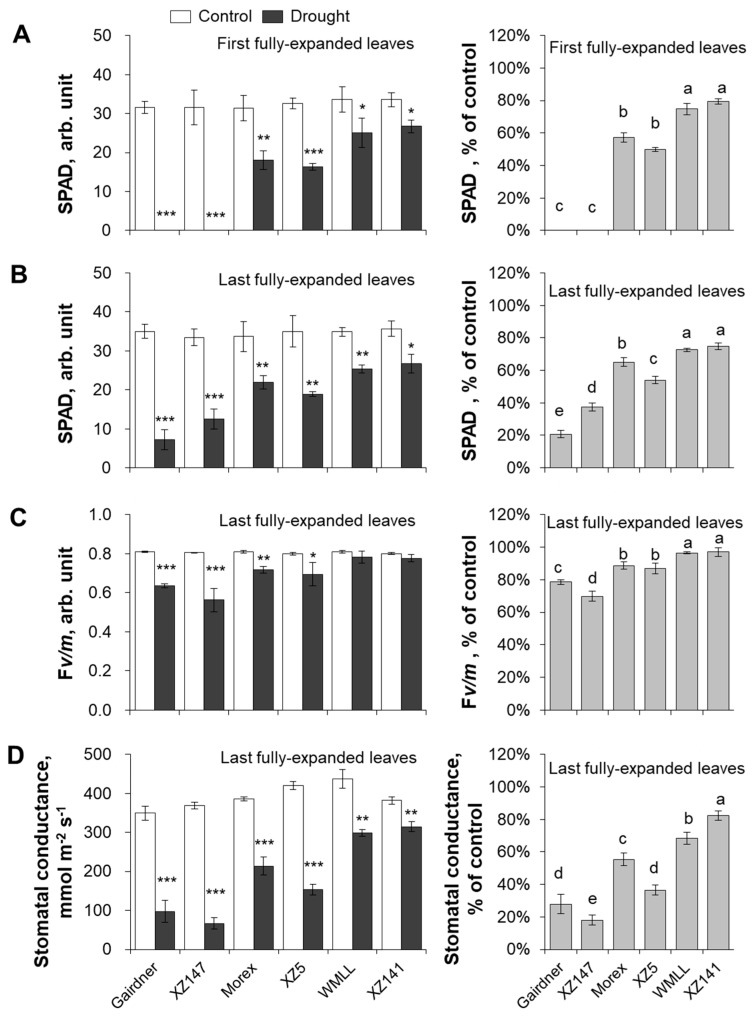
Effect of water-limited drought stress on photosynthetic parameters of barley seedlings. Twelve-day-old barley seedlings were subjected to drought stress. Plants were measured for SPAD values of the first (**A**) and last (**B**) fully-expanded leaves, F*v/m* of last fully-expanded leaf (**C**) and stomatal conductance of last fully-expanded leaf (**D**) at 10 d after onset of treatments. Vertical bars represent means ± SE of five replicates. Asterisks indicate the significant difference between treatments at * *p* < 0.05, ** *p* < 0.01 and *** *p* < 0.001. Different lowercase letters indicate the significant difference between genotypes at *p* < 0.01.

Photosynthesis is the most fundamental and intricate physiological process in plants, and is severely affected by drought stress [35]. In the present study, the impact of drought stress on the photosynthetic ability was investigated by using six barley genotypes contrasting in drought tolerance in terms of chlorophyll content (SPAD value), maximum quantum yield of PSII (chlorophyll fluorescence F*v/m* ratio) and stomatal conductance (*gs*). After 10 days of drought stress (stopping of watering), SPAD values of both the first and the last fully-expanded leaves were significantly reduced by a range of 20%–100% (Figure 2A,B). The first fully-expanded leaves were affected more than the last fully-expanded ones, with 5% to 40% more reduction observed for the former (Figure 2A,B). The drought-induced decline in chlorophyll content was genotypic-specific. Of the six barley genotypes, WMLL and XZ141 maintained the highest chlorophyll content under drought stress (ca. 73% to 80% of the control), followed by Morex and XZ5 (ca. 50% to 65% of the control). Gairdner and XZ147 showed a much higher sensitivity to drought conditions than the above four genotypes, with only 0 (totally wilt) to 37% SPAD value of the control maintained (Figure 2A,B). The maximum photochemical efficiency of PSII (chlorophyll fluorescence F*v/m* value) from the last fully-expanded leaf was also significantly affected by drought stress in the sensitive or moderate tolerant barley genotypes (Figure 2C). Their F*v/m* values were greatly reduced by a range of from 11% (in Morex) to 30% (in XZ147). However, no significant impact was observed on the chlorophyll fluorescence of the tolerant WMLL and XZ141 after drought stress (Figure 2C). A substantial decline in stomatal conductance (*gs*) was found in all six barley genotypes, ranging from 18% to 82% (Figure 2D). Similarly, the tolerant WMLL and XZ141 showed the highest *gs* values (ca. 68% and 82% of the control, respectively), followed by the moderate tolerant Morex and XZ5 (ca. 55% and 36% of the control, respectively). The sensitive Gairdner and XZ147 performed the worst, with only 28% and 18% of their *gs* values maintained after drought stress, in comparison to the control (Figure 2D).

### 2.2. Genotypic Difference in Drought Sensitivity is Highly Dependent on the Ability of Water Retention by Osmotic Adjustment 

**Figure 3:**
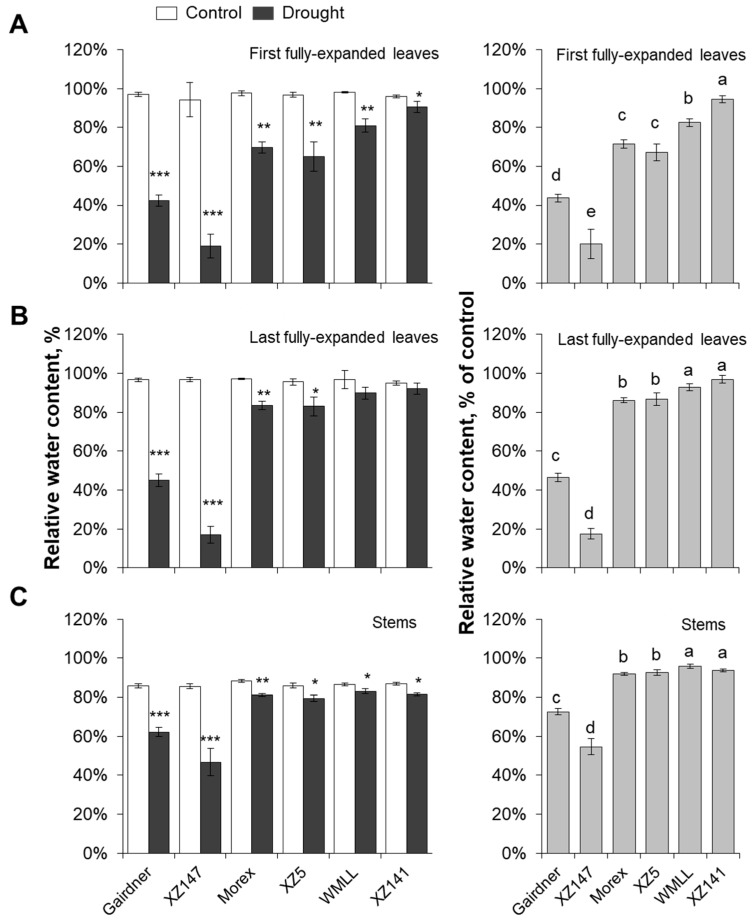
Effect of water-limited drought stress on water relation of barley seedlings. Twelve-day-old barley seedlings were subjected to drought stress. Plants were harvested for relative water content analysis in first (**A**) and last (**B**) fully-expanded leaves and stem (**C**) at 10 d after onset of treatments. Vertical bars represent means ± SE of five replicates. Asterisks indicate the significant difference between treatments at * *p* < 0.05, ** *p* < 0.01 and *** *p* < 0.001. Different lowercase letters indicate the significant difference between genotypes at *p* < 0.01.

**Figure 4:**
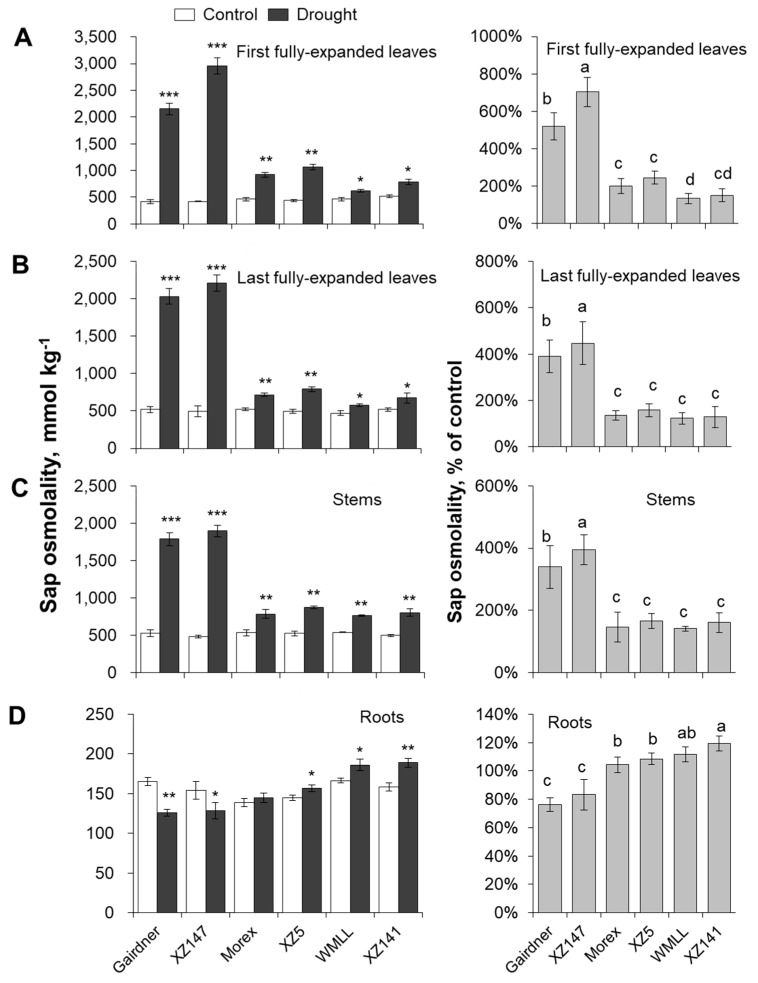
Effect of water-limited drought stress on osmotic adjustment of barley seedlings. Twelve-day-old barley seedlings were subjected to drought stress. Plants were harvested for osmolality analysis in first (**A**) and last (**B**) fully-expanded leaves, stem (**C**) and root (**D**) at 10 d after onset of treatments. Vertical bars represent means ± SE of five replicates. Asterisks indicate the significant difference between treatments at * *p* < 0.05, ** *p* < 0.01 and *** *p* < 0.001. Different lowercase letters indicate the significant difference between genotypes at *p* < 0.01.

Relative water content (RWC) in the leaves and stems were significantly decreased in response to drought stress (Figure 3). On average of the six barley genotypes, the impact of drought stress was much more severe on the first-expanded leaf than the last-expanded leaf and stem (Figure 3). A significant difference in RWC between genotypes was observed. Gairdner and XZ147 suffered the most severe decline in RWC among the six barley genotypes, with 58% and 83% reduction for the first-expanded leaf, 55% and 81% reduction for the last-expanded leaf and 38% and 53% reduction for stem respectively (Figure 3). On the other hand, RWC in the first-expanded leaves and stems of WMLL and XZ141 were only slightly reduced (Figure 3A,C), and their last-expanded leaves were not significantly changed by drought (Figure 3B). To a large extent, changes in RWC were mirrored by changes in sap osmolality (Figure 4A–C). Drought stress resulted in a massive increase in the sap osmolality of leaves and stems. On average of the six barley genotypes, the first-expanded leaves demonstrated the largest increase in sap osmolality (ca. 3.3-fold, Figure 4A), followed by the last-expanded leaves (ca. 2.3-fold, Figure 4B) and stems (ca. 2.2-fold, Figure 4C). Gairdner and XZ147 showed a clear difference from the other four barley genotypes, with an approximately 2.4- to 4.5-fold higher sap osmolality in their leaves and stems (Figure 4A–C). The effect of drought stress on root sap osmolality was highly dependent on the tolerance of barley genotypes. Similar to the leaves and stems, the sap osmolality of roots in the tolerant WMLL and XZ141 were significantly increased after an onset of drought stress, whereas those in the sensitive genotypes Gairdner and XZ147 were significantly decreased (Figure 4D).

### 2.3. Drought Tolerance is Highly Correlated with the Ability of K^+^ Uptake and Accumulation in Barley

**Figure 5:**
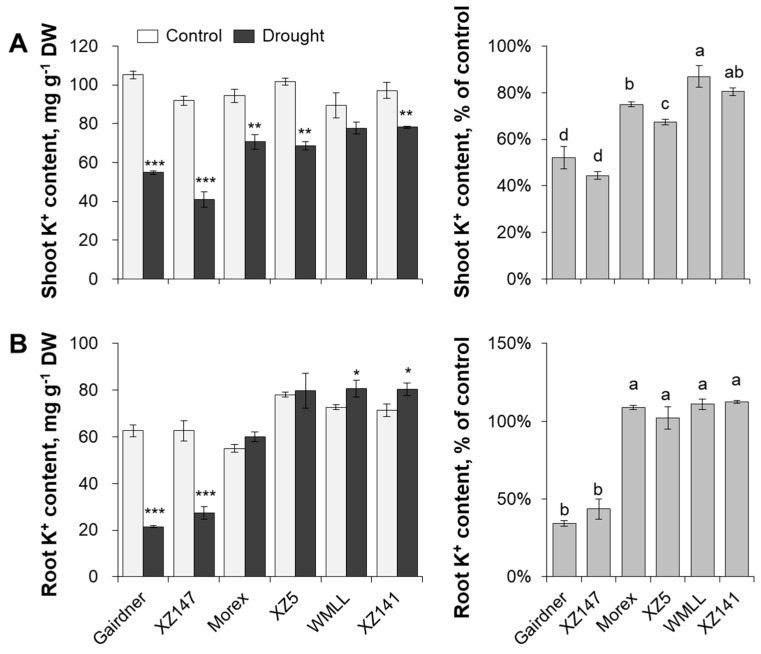
Effect of water-limited drought stress on tissue K^+^ contents of barley seedlings. Twelve-day-old barley seedlings were subjected to drought stress. Plants were harvested for K^+^ contents in shoot (**A**) and root (**B**) at 10 d after onset of treatments. Vertical bars represent means ± SE of five replicates. Asterisks indicate the significant difference between treatments at * *p* < 0.01, ** *p* < 0.01 and *** *p* < 0.001. Different lowercase letters indicate the significant difference between genotypes at *p* < 0.01.

**Figure 6:**
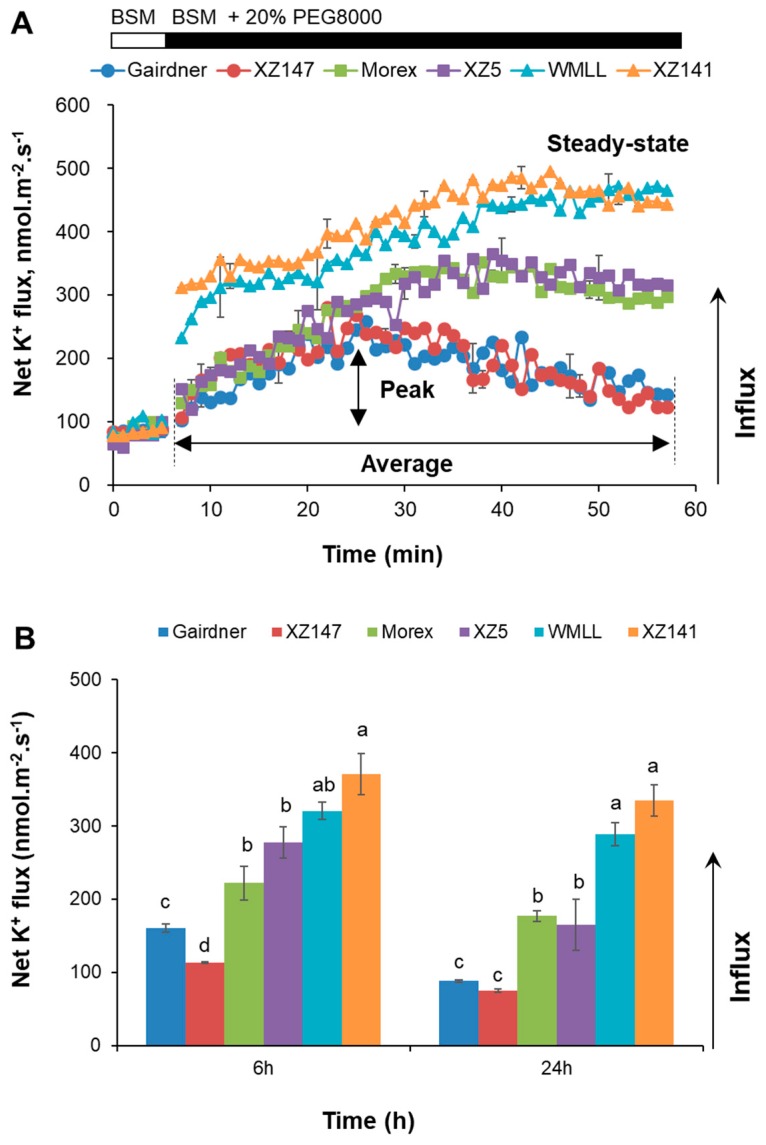
Transient (**A**) and steady-state (**B**) net K^+^ flux response to drought stress generated by 20% PEG8000 measured from mature (∼20 mm from root tip) zones of six barley varieties contrasting in drought stress tolerance. Data are mean ± SE (*n* = 6 individual plants). The onset of treatment is indicated by an arrow. Measurements were undertaken at room temperature (24 ± 1 ℃). Different lowercase letters indicate the significant difference between genotypes at *p* < 0.01.

The capabilities of root K^+^ uptake and root to shoot K^+^ translocation were then compared between the contrasting barley genotypes in terms of the tissue K^+^ contents and root epidermis net K^+^ fluxes (Figure 5 and Figure 6). Drought stress resulted in a significant decline in shoot K^+^ content, with a range from 13% to 56% depending on the genotype (Figure 5A). This decline was much more severe in the drought-sensitive genotypes (Gairdner and XZ147) than in the drought-tolerant ones (WMLL and XZ141). The effect of drought stress on root K^+^ content was very similar to that on root sap osmolality. Root K^+^ contents in the sensitive Gairdner and XZ147 were significantly decreased after onset of drought stress, whereas those in the tolerant WMLL and XZ141 were significantly increased (Figure 5B). 

The capability of K^+^ uptake by barley roots were further investigated in terms of net K^+^ fluxes using the MIFE technique. A strong net K^+^ uptake of 77 to 92 nmol m^−2^ s^−1^ (depending on genotype) was measured from the mature root epidermis of all barley genotypes in the control (Figure 6A). The application of 20% PEG8000 resulted in a significant increase in K^+^ influx. In the sensitive genotypes Gairdner and XZ147, this increase was short-lived, and net K^+^ influxes returned to their steady-state levels within 50 min of the stress onset (Figure 6A). In the tolerant genotypes WMLL and XZ141, however, net K^+^ influxes were rapidly increased after onset of stress and saturated around 450 nmol m^−2^ s^−1^ (Figure 6A). The moderate tolerant genotypes Morex and XZ5 demonstrated a similar responding trend in net K^+^ fluxes to that in the tolerant ones, but with a smaller magnitude of net K^+^ influxes (around 300 nmol m^−2^ s^−1^). With the exposure time of drought stress increasing to 24 h, net K^+^ influxes of both moderate (Morex and XZ5) and tolerant (WMLL and XZ147) barley genotypes were also recovered to some extent (Figure 6B). However, their net K^+^ influxes maintained much more than the sensitive genotypes (Figure 6B; *p* < 0.01). All these results indicated that the tolerance to drought is significantly correlated with the ability of K^+^ uptake and accumulation in barley (Figure S1, *p* < 0.01).

### 2.4. Drought Tolerant Barley Genotypes are Capable of Maintaining H^+^-Pumping Activity 

**Figure 7:**
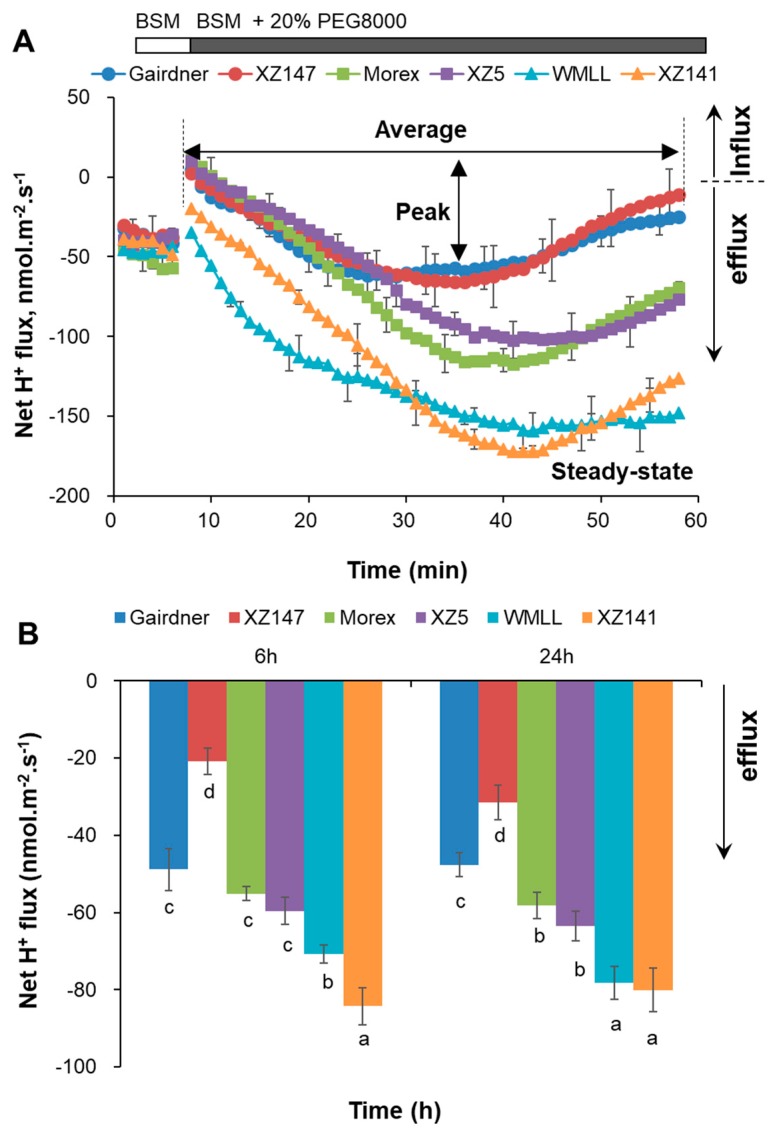
Transient (**A**) and steady-state (**B**) net H^+^ flux response to drought stress generated by 20% PEG8000 measured from mature (∼20 mm from root tip) zones of six barley varieties contrasting in drought stress tolerance. Data are mean ± SE (*n* = 6 individual plants). The onset of treatment is indicated by an arrow. Measurements were undertaken at room temperature (24 ± 1 ℃). Different lowercase letters indicate the significant difference between genotypes at *p* < 0.01.

Significant H^+^ effluxes were observed from the mature root epidermis of all six barley genotypes in the background of BSM solution (Figure 7A), suggesting a high H^+^-ATPase pumping activity owned by these cells under the control condition. After onset of 20% PEG treatment, such H^+^ effluxes were dramatically increased, but differed greatly between the contrasting barley genotypes (Figure 7). The peak values of PEG-induced H^+^ effluxes were around −60 nmol m^−2^ s^−1^, −110 nmol m^−2^ s^−1^ and −166 nmol m^−2^ s^−1^ for the sensitive, moderate and tolerant genotypes, respectively. Furthermore, net H^+^ effluxes of the sensitive Gairdner and XZ147 started to recover within 25 min of the stress onset and returned to the control level at 50 min. However, net H^+^ effluxes of the moderate and tolerant genotypes started to recover much later than those of the sensitive ones, and were unable return to the control level after 50 min of the stress onset, which consequently led to a significant difference in the final steady-state net H^+^ efflux between the contrasting genotypes at the end of the measurement (Figure 7A). With the exposure time increasing to 6 h, net H^+^ effluxes of both the moderate and tolerant genotypes decreased further as compared to the end of the transient flux measurement, and maintained around −58 to −80 nmol m^−2^ s^−1^ until 24 h after the stress onset (Figure 7B). Surprisingly, net H^+^ effluxes of the sensitive genotypes increased by 90% at 6 h of the stress onset as compared to the end of the transient flux measurement, on average of two genotypes (Figure 7B). Subsequently, net H^+^ efflux of Gairdner was maintained around −48 nmol m^−2^ s^−1^ until 24 h after the stress onset, whereas net H^+^ efflux of XZ147 further increased by 50% as compared to the value at 6 h (Figure 7B).

### 2.5. Drought Stress Induces Changes in Relative Expression of Plasma Membrane K^+^ Transporting and H^+^ Pumping-Related Genes

**Figure 8:**
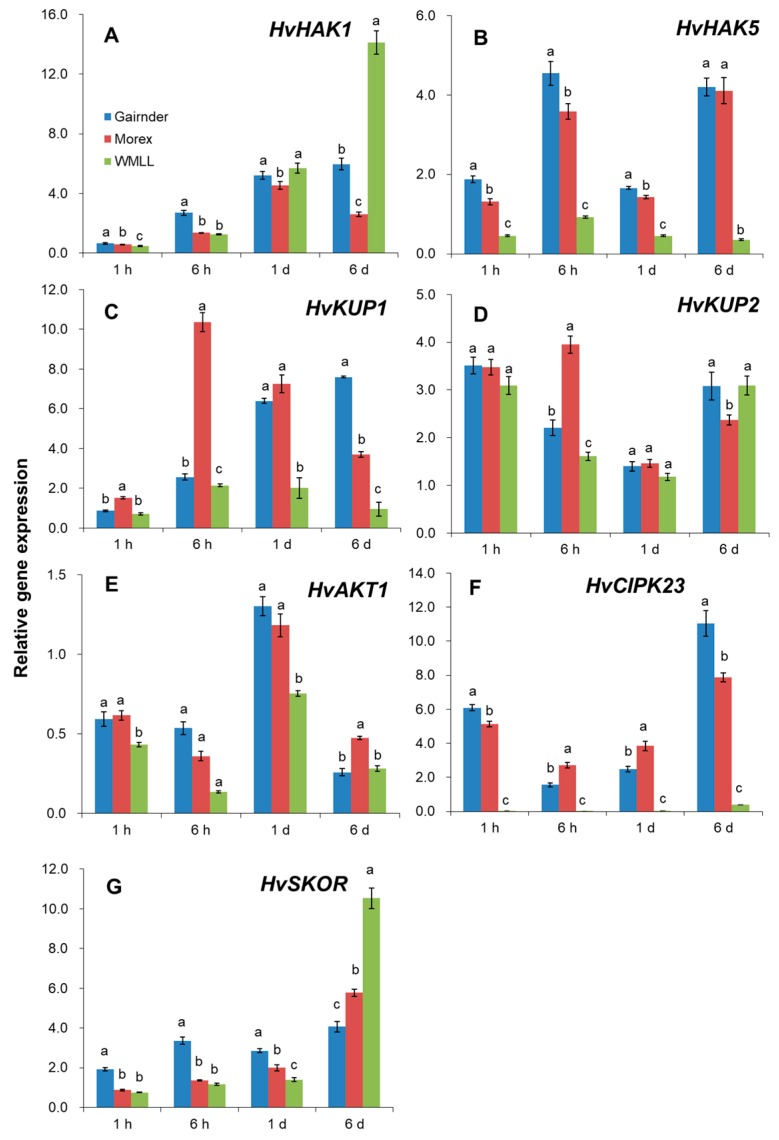
Transcript levels of genes conferring K^+^ uptake [*HvHAK1* (**A**), *HvHAK5* (**B**), *HvKUP1* (**C**), *HvKUP2* (**D**), *HvAKT1*(**E**) and *HvCIPK23* (**F**)] and xylem loading [*HvSKOR* (**G**)] in barley roots after onset of drought stress generated by 20% PEG8000. Gene expression under control condition at each period was normalized to 1, and gene expression under drought condition at every sampling time was compared relative to it. Vertical bars represent means ± SE of three biological replicates. Different lowercase letters indicate the significant difference between genotypes at *p* < 0.01.

The relative expression pattern of genes conferring K^+^ uptake (*HvHAK1*, *HvHAK5*, *HvKUP2* and *HvAKT1*) and xylem loading (*HvSKOR*) in roots were further compared across three contrasting barley genotypes (sensitive Gairdner, moderate Morex and tolerant WMLL) to find out which of these genes are contributing to the genotypic difference in K^+^ uptake and accumulation under drought stress. The relative transcript levels of all these genes were significantly affected by drought stress (generated by 20% PEG8000). HAK1, HAK5, KUP1 and KUP2, as members of KUP/HAK/KT transporter family, are crucial for the high-affinity K^+^ transport in plants [36,37,38]. In the present study, the expression of these genes changed greatly in responding to drought stress and performed in a genotyping-dependent matter (Figure 8A–D). The expression of *HvHAK1* was reduced approximately by half after drought treatment for 1 h, but dramatically increased afterwards (Figure 8A). Notably, the expression of *HvHAK1* in WMLL was induced as much as 14-fold compared to the control value, which was significantly higher than that in Gairdner and Morex (Figure 8A). The expression of *HvHAK5* was highly induced by drought stress even under the short-term treatment (ca. 1 h) in Gairdner and Morex, whereas it was deeply suppressed in drought tolerant genotype WMLL during the entire exposure time of drought stress (Figure 8B). The expression of *HvKUP1* was not changed much after the first 1 h of the drought stress, but differed greatly between the three barley genotypes with the exposure time increasing (Figure 8C). The expression of *HvKUP1* in Gairdner was gradually induced within the duration of drought, and reached a 7.6-fold expression of the control at 6 d. In Morex, the expression of *HvKUP1* was dramatically induced by drought and peaked at 6 h by reaching a 10-fold expression of the control. However, it was slightly induced in WMLL after 6 h of the treatment, falling back to the control level at 6 d again. Unlike the above genes, the expression of *HvKUP2* was dramatically induced by drought stress within 1h and then constantly maintained an up-regulative expression compared to the control in all genotypes (Figure 8D). Taken together, the response of HAK/KUP family members to drought stress was in a time- and genotype-specific manner, which consequently led to a significant difference in their root K contents (Figure 5B and Figure S2B).

AKT1 and SKOR are members of the Shaker-type potassium channel family, which greatly contribute to the low-affinity uptake and xylem loading of K^+^ in plant roots, respectively [39,40]. After onset of drought stress, surprisingly, the expression of *HvAKT1* were changed little or even dramatically reduced in all barley genotypes (Figure 8E). Moreover, the transcript level of *HvAKT1* in WMLL was notably lower than in Gairdner and Morex during the entire exposure time of PEG treatment (Figure 8E). These results indicated that the change in the transcript level of *HvAKT1* might not confer the genotypic difference in drought tolerance in barley. Therefore, the expression of *HvAKT1* regulator gene, *HvCIPK23*, was further investigated. Our results showed that the transcript level of *HvCIPK23* was dramatically increased by drought stress within 1 h and constantly maintained an up-regulative expression compared to the control in Gairdner and Morex (Figure 8F). In the tolerant genotype WMLL, however, the transcript level of *HvCIPK23* was too low to be detected at either time of drought onset (Figure 8F). On the other hand, the expression of *HvSKOR* was increased by 90% in the sensitive genotype Gairdner after 1 h of treatment onset, and maintained 1.9 to 3.1-fold increase till 6 d after the drought stress (Figure 8G). Although the expression of *HvSKOR* in the moderate Morex and tolerant WMLL were much lower than in the sensitive Gairdner, they were greatly increased by 4.8- and 9.5-fold respectively, which consequently resulted in a significantly higher K^+^ content in their shoots than that of Gairdner (Figure 5A and Figure S2A). 

**Figure 9:**
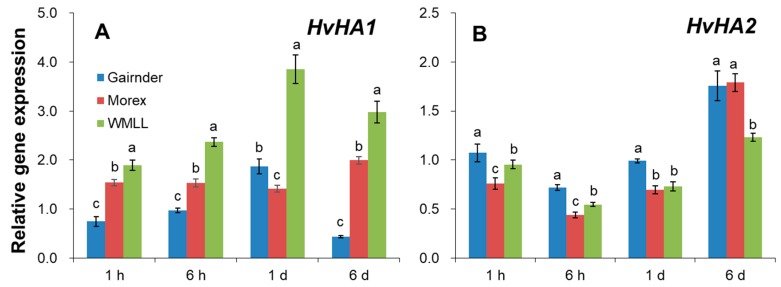
Transcript levels of plasma membrane H^+^-ATPase genes *HvHA1* (**A**) and *HvHA2* (**B**) in barley roots after onset of drought stress generated by 20% PEG8000. Gene expression under control condition at each period was normalized to 1, and gene expression under drought condition at every sampling time was compared relative to it respectively. Vertical bars represent means ± SE of three biological replicates. Different lowercase letters indicate the significant difference between genotypes at *p* < 0.01.

The results of net ion fluxes in response to drought stress showed that the great drought-induced K^+^ influxes were mirrored with the substantial H^+^ effluxes, and the magnitude of these H^+^ effluxes were highly dependent on barley genotypes’ tolerance of drought stress (Figure 6 and Figure 7). Therefore, the expression of the genes conferring plasma membrane (PM) H^+^-ATPase (*HvHA1* and *HvHA2*) in response to drought stress were further examined (Figure 9). The expression of *HvHA1* showed a very different pattern in the three contrasting genotypes (Figure 9A). In the sensitive genotype Gairdner, the expression of *HvHA1* was first reduced at 1 h of the drought stress, then gradually increased to 1.87-fold of the control at 1 d of the treatment, and again decreased to 44% of the control at 6 d (Figure 9A). In the moderate and tolerant genotypes Morex and WMLL, however, the expression of *HvHA1* were highly upregulated within 1 h of drought stress, and maintained such high expression levels during the entire exposure time (Figure 9A). This regulation in the expression of *HvHA1* nicely matched the reported increase in net H^+^ efflux measured by MIFE (Figure 7). On the other hand, the expression of *HvHA2* showed a comparable pattern in all three genotypes, showing little change at 1 h, reducing by 27% to 55% at 6 h and 1 d, and then increasing by 22% to 78% at 6 d of drought stress (Figure 9B). It is noteworthy that the expression level of *HvHA2* in WMLL was significantly lower (1.5-fold) than those in Gairdner and Morex at 6 d of stress onset.

## 3. Discussion

### 3.1. K^+^ Uptake Confers Osmotic Adjustment and Contributes to Genotypic Difference in Drought Tolerance in Barley

Drought stress imposed at seedling stage severely hampered the growth of barley plants, as characterized by the drastic reduction of shoot dry mass (Figure 1), which can be mainly attributed to the disruption of water relations and leaf photosynthetic properties [41,42] (Figure 2 and Figure 3). Furthermore, the drought-induced reduction in barley plant growth differed greatly among barley genotypes, which was highly correlated with their significant difference in the ability to adjust the osmolality in roots and leaves (Figure 4). It has been documented that K^+^ contributes between 35% and 50% on average of the cell osmotic potential in crops [43,44]. Growing evidence shows that plants have a larger internal requirement for K^+^ in response to drought stress [45]. It has been proven that the ability to retain K^+^ homeostasis is one of the key traits conferring the broad range of plant tolerance to abiotic stresses, such as salinity and waterlogging [38,44,46,47,48,49,50]. Early studies in our laboratory have also indicated that the ability of K^+^ uptake may be involved in the drought tolerance of barley [34]. This hypothesis is now fully validated by investigating more barley cultivars differing in drought tolerance. We showed that the whole-plant performance of six contrasting barley genotypes under drought conditions (Figure 1) correlated with tissue K^+^ contents (Figure 2 and Figure 5) and root net K^+^ influxes (Figure 6). Moreover, the inability of roots to effectively uptake and translocate K^+^ resulted in a failure in osmotic adjustment in sensitive genotypes (Figure 4), which thereby disrupted their water relation (Figure 2) and consequently hampered the plant growth (Figure 1). Taken together, it might be suggested that the root K^+^ uptake and root to shoot K^+^ translocation are key determinants of plant adaptive ability to drought stress.

### 3.2. Regulation of the K Transporters and Channels is Critical for Root K^+^ Uptake and Root to Shoot K^+^ Translocation under Drought Stress in Barley

Drought-induced changes in root K^+^ uptake and root to shoot K^+^ translocation may be triggered by the regulation of a series of K^+^-specific channels and transporters [51]. In plants, K^+^ uptake from the external environment is mainly mediated by the low-affinity voltage-dependent K^+^-inward (KIR) rectifying channels such as AKT1 under normal K^+^ condition, and the high-affinity transporters from HAK/KUP/KT family under K^+^ deficiency [37,39,41]. Considering their crucial role in K^+^ uptake, it can be hypothesized that more upregulation of AKT1 and HAK/KUP/KT family members may confer higher drought tolerance in barley. However, the present study demonstrated otherwise. Our results showed that the expression of *HvAKT1* was deeply suppressed by drought stress and was much lower in the tolerant barley (Figure 8D). In this context, the current results are consistent with a previous study which reported that the disruption of *AKT1* conferred an enhanced response to water stress, with much lower transpiration and less water consumption in *akt1* mutants than in the wild type [52]. These results indicated that the high transcript level of *HvAKT1* might not confer high drought tolerance in barley. It has been well documented that AKT1-mediated K^+^ uptake is modulated by CBL1/9-CIPK23 complex and CBL1-CIPK23 complex in *Arabidopsis* and rice roots, respectively [53,54]. In the present study, the transcript level of *HvCIPK23* was dramatically increased by drought stress in Gairdner and Morex (Figure 8F). In the tolerant genotype WMLL, however, it was too low to be detected at either time of drought onset (Figure 8F). It can be hypothesized that the activation of AKT1 by CIPK23 is critical for the adaptive response to drought stress in the sensitive barley genotypes, but it is not the case in the drought tolerant barley. 

The HAK/KUP/KT family is the largest K^+^ transporter family mediating K^+^ transport across the plasma membrane [55], and several members of the family have been verified to act positively in osmotic adjustment by maintaining potassium homeostasis in *Arabidopsis* [33]. Recently, HAK5 was found to be promoted by INTEGRIN-LINKED KINASE1 (ILK1) and to positively regulate osmotic stress tolerance in *Arabidopsis* [56]. Chen et al. [57] have also proved that *OsHAK1* enhances drought tolerance in rice through systemic regulation of potassium homeostasis and activation of stress-related genes. In the present study, the gene expression of *HvHAK1*, *HvHAK5*, *HvKUP1* and *HvKUP2* were investigated under drought conditions. Our results showed that the expression of these transporter genes were responding to drought stress in a time- and genotype-specific manner (Figure 8A–D). At the initial time of drought onset (ca. 1 h in the present study), the expression of *HvHAK1* and *HvKUP1* showed little change, whereas the expression of *HvHAK5* and *HvKUP2* significantly increased compared to the control condition (Figure 8A–D). With the increase of drought duration (ca. 6 d in the present study), the expression of *HvHAK1* and *HvKUP1* were highly increased, whereas the expression of *HvHAK5* and *HvKUP2* still maintained an up-regulative trend compared to the control condition (Figure 8A–D). These results suggested that *HvHAK1*, *HvHAK5*, *HvKUP1,* and *HvKUP2* are all critical for K^+^ uptake under drought stress, but their responses to drought stress initiate at different times, showing a quick response of HAK5 and KUP2 as opposed to a slow but lasting response of HAK1 and KUP1. Furthermore, the expression of *HvHAK1*, *HvHAK5*, *HvKUP1,* and *HvKUP2* in response to drought stress differed greatly between barley genotypes contrasting in drought tolerance. Compared to the sensitive and moderate genotypes Gairdner and Morex, although the tolerant genotype WMLL showed a much lower expression of *HvHAK5* and *HvKUP1*, it had a much higher expression of *HvHAK1* under the drought stress, which might consequently lead to a significant difference in their root K contents (Figure 5B and Figure S2B). These results indicated that different barley genotypes preferred different K^+^ transporters to uptake K^+^ under drought stress. To further elucidate such genotypic preferring, the gene expression of more K^+^ transporters under drought stress needs to be investigated using a large-scale of genotypes.

Stelar K^+^ outward rectifier channel (SKOR), known to be expressed at the xylem parenchyma interface [58], is one crucial Shaker-type channel that mediates K^+^ translocation from root to the shoot [59]. Gaymard et al. [40] found that the disruption of *SKOR* severely hampers xylem K^+^ loading and consequently results in a dramatic decrease in shoot K^+^ content. It is also involved in plant adaptive response to drought stress. However, the reported experimental results remain controversial. In *Arabidopsis*, *SKOR* expression is suppressed by drought stress conditions [60]. In *Zygophyllum xanthoxylum*, however, *ZxSKOR* was induced under osmotic stress and improved drought tolerance of the plants [61]. In the present study, the expression of *HvSKOR* differed greatly between the contrasting barley genotypes. In the sensitive genotype Gairdner, it was significantly increased after onset of drought stress (Figure 8G), coinciding with the previous study on *Zygophyllum xanthoxylum*. However, in the moderate Morex and tolerant WMLL, the expression of *HvSKOR* was kept at a very low level under the short-term drought stress, indicating its role in entire plant potassium homeostasis and osmotic adjustment under drought condition [62]. Conversely, upon a long-term drought stress, the expression of *HvSKOR* was highly induced in these two genotypes, which consequently led to significantly higher shoot K^+^ contents in Morex and WMLL than in Gairdner (Figure 5A and Figure S2A), suggesting an important role of SKOR in maintaining root to shoot K^+^ translocation under drought conditions. 

### 3.3. Plasma Membrane H^+^-ATPase Activity and/or Expression Plays an Important Role in Regulating the Activity of K Transporters and Channels under Drought Stress

Plasma membrane (PM) H^+^-ATPases are the primary pumps responsible for establishing cellular membrane potential and electrochemical H^+^ gradients, which is essential for plant nutrient acquisition and partitioning [62,63]. In the present study, net H^+^ efflux was induced by drought stress and maintained much higher steady-state values in the moderate and tolerant genotypes than in the sensitive ones. All these results suggested that the H^+^-pumping activity plays an important role in barley roots’ response to drought stress, and the tolerant barley genotypes are capable of maintaining a higher level of H^+^-pumping activity than the sensitive ones under drought stress. Furthermore, higher net K^+^ influxes under drought conditions were observed, accompanied by the larger effluxes of H^+^ in a very clear and genotype-specific manner (Figure 6 and Figure 7), suggesting an important role of PM H^+^-ATPases in regulating transmembrane K^+^ transport. PM H^+^-ATPases consist of a large gene family in plants, with 11 members in *Arabidopsis* (AHA1-11). AHA1 and AHA2 are found to express in almost all tissues and organs, functioning as housekeeping genes required for ion homeostasis [64]. Single knockout of *AHA1* or *AHA2* has no apparent phenotypes while double knockout is lethal [65,66], indicating the compensation roles between AHA1 and AHA2 as well as the key roles they have for normal plant metabolism. Complex temporal modulation of H^+^-ATPase activity was reported during early pathogen recognition events [63]. Similarly, temporal modulation of H^+^-ATPase was also observed in the present study. The induction of *HvHA1* was observed after drought stress treatment for 1 h in Morex and WMLL, and for 6 h in Gairdner (short-term drought stress, Figure 9A), indicating a quick response of *HvHA1* to drought stress. However, the induction of *HvHA2* was not detected until the exposure time of drought treatment increased to 6 d (long-term drought stress; Figure 9B). These results demonstrated that *HvHA1* and *HvHA2* respond to drought stress in a time-specific manner, although they have expression overlap and functional redundancy. Notably, the drought-induced expression of *HvHA1* nicely matched the reported increase in net H^+^ efflux measured by MIFE, suggesting a determinant role of *HvHA1* in regulating ion homeostasis in drought stressed barley roots. In this context, the current results are consistent with a previous study of wheat which reports a dramatic increase in PM H^+^-ATPase activity in response to drought condition [67]. In addition, the drought tolerant genotype WMLL showed the highest H^+^ efflux under drought stress (Figure 6), while its expression level of *HvHA2* was much lower than the sensitive genotype Gairdner (Figure 9B), indicating that besides transcriptional regulation, post-transcriptional regulation of HvHA2 also matters in response to drought stress [68,69]. Indeed, it has been reported in tomato cells that H^+^-ATPases could be activated in response to osmotic stress through the formation of a membrane 14-3-3/H^+^-ATPase complex [70]. Taken together, the data reported in the present study implicate that PM H^+^-ATPase activity and/or expression plays an important role in regulating the K^+^ transport across PM under drought stress.

## 4. Materials and Methods 

### 4.1. Plant Materials, Growth Conditions and Treatments

Six barley (*Hordeum vulgare L.*) genotypes, namely Gairdner (Australian modern malting barley cultivar), Morex (American modern malting barley cultivar), Wumangliuleng (WMLL, Chinese landrace barley), XZ5, XZ141, and XZ147 (Tibetan annual wild barley) were used in this study. Among these barley genotypes, Gairdner and XZ147 are sensitive, Morex and XZ5 are moderately tolerant, and WMLL and XZ141 are tolerant to drought, respectively (unpublished data). Seeds were multiplied in the field of Zhejiang University, Hangzhou. Seeds were surface-sterilized for 15 min with 10% commercial NaClO (0.52% final concentration of active Cl) and then rinsed for 30 min with tap water. Sterilized seeds were germinated in the dark at 26 ℃ for 48 h in the controlled growth chamber. Uniform well-germinated seeds were then planted in the vermiculite and grown with one-fifth Hoagland solution (pH6.0) [71] in the controlled growth room with a photoperiod of 16/8 h, light intensity of 200 ± 25 µmol m^−2^ s^−1^, temperature of 22/18 ℃ (day/night), and relative humidity of 60%. Barley seedlings were grown to have two fully-expanded leaves (for 12 days) prior to the drought treatment, in order to make the difference in the phenotypic performance as significant as possible between treatments and barley genotypes. Thereafter, irrigation was stopped for half the barley seedlings to generate a drought condition, whilst the others were well irrigated to the water content of 40% (HH2 Moisture Meter, Delta-T Devices, Cambridge, UK). Ten days later, shoots and roots of barley plants under both control (water content of ∼40%) and drought (water content of ∼4%) conditions were sampled to investigate the morphological performance as well as the physiological parameters.

### 4.2. Biomass and Ratio of Wilting Leaves

Prior to the harvest of the biomass, the number (no.) of wilting leaves and the total number leaves from each of the drought-stressed plants were counted. The leaves that were ≤ 50% wilted were counted as 0.5 and those that were > 50% wilted as 1. The ratio of wilting leaves was then calculated according to the equation: ratio of wilting leaves = no. of wilting leaves/no. of total leaves. The shoot of the counted plant was then excised, dried at 75 ℃ in the oven for 2 days, and weighted for the dry weight. Five replicates were randomly taken for each barley genotype.

### 4.3. SPAD, Chlorophyll Fluorescence and Stomatal Conductance

Leaf chlorophyll content was measured from both the first and the last fully-expanded leaves with a SPAD meter (SPAD-502 Plus, Konica Minolta, Inc. Tokyo, Japan). Chlorophyll fluorescence was measured from the latest fully-expanded leaf with a portable fluorimeter (OS-30p+, Opti-Sciences, Inc. Hudson, NH, USA). Plants were dark-adapted for 30 min prior to measurement. The maximum quantum efficiency of photosystem II (F*v*/*m* = (F*m*–F*o*)/F*m*) was recorded at a saturating actinic light (660 nm) with an intensity of 1100 µmol m^−2^ s^−1^. Stomatal conductance was measured from the latest fully-expanded leaf with a porometer (Leaf Porometer SC-1, Decagon Devices, Inc. Pullman, WA, USA). All three measurements were conducted on the middle part of the fully-expanded leaves following the manufacturer’s instructions. Ten replicates were randomly taken for each barley genotype under either control or drought condition.

### 4.4. Relative Water Content (RWC)

Fresh leaves from plants under control or drought condition were collected and weighed immediately for their fresh weight (FW). The weighed leaves were then soaked in deionized water for 2 h at room temperature, and their turgid weights (TW) was weighed subsequently. Dry weights (DW) were determined after drying for 2 days at 75 ℃. RWC was calculated using the following formula: RWC% = (FW − DW) / (TW − DW) × 100%. Five replicates were determined for each barley genotype under either control or drought conditions.

### 4.5. Sap Osmolality 

After harvesting, the first and last fully-expanded leaves, stems, and roots were collected separately and immediately stored in the 1.5 mL centrifuge tubes at −20 ℃. Tissue sap was extracted through the freeze-thawing method according to Tomos et al. [72]. After centrifuged at 10,000 g for 3 min, the extracted sap was examined for its osmolality using a vapor pressure osmometer (Vapro 5600; Wescor Inc. Logan, UT, USA).

### 4.6. K^+^ Content 

Dried barley leaves or roots were weighed and digested with HNO_3_ at 120 ℃ for 2 h. The digested solution was then diluted with deionized water and analyzed for K^+^ concentration using atomic absorption spectrophotometry (AA6300, Shimadzu, Japan). Tissue K^+^ contents were then calculated with the K^+^ concentrations in the diluted solution and tissue dry weights.

### 4.7. Ion flux Measurements

Net K^+^ and H^+^ fluxes were measured using the MIFE technique (University of Tasmania, Hobart, Australia), essentially as described in our previous publications [73,74]. Barley seedlings were grown in the dark at 25 ± 1 ℃ with aerated basic salt medium (BSM, 0.5 mM KCl + 0.1 mM CaCl_2_; pH was adjusted to 6.0 with HCl or KOH). Three-day seedlings (with only ∼ 1 cm long coleoptile) with 7 ~ 8 cm long roots, which can be easily handled and immobilized in the measuring chamber, were used for ion flux measurements. Ion-selective microelectrodes containing specific liquid ion exchanger (LIX; K^+^ 60031 and H^+^ 95297, Sigma-Aldrich Chemie GmbH, St. Louis, WA, USA) in the tips were positioned 50 µm above the root surface. Ion fluxes were measured by a slow (6 s half-cycle) square-wave movement of electrodes between two positions, close to (50 µm) and away from (100 µm) the root surface. Net ion fluxes were measured from the mature root (∼20 mm from the root tip) epidermis. The voltage difference between two positions was recorded by the MIFE CHART software and converted to the electrochemical potential difference using the calibrated Nernst slope of the electrodes. Net ion fluxes were then calculated from the electrochemical potential difference using cylindrical diffusion geometry with the MIFEFLUX program [75,76]. 

For the transient ion flux measurements, roots of intact seedlings were horizontally immobilized on a glass slide in a petri dish containing BSM 1 h prior to the measurement. Transient ion fluxes were measured for 5 min under the control condition (BSM solution, pH 6.0) as the steady initial net fluxes. Subsequently, BSM solution was replaced by 20% PEG8000 in the background of BSM solution, and transient ion flux responses were recorded for another 50 min. The period of time for changing the solution (∼2 min) was discarded from the data analysis. Eight individual roots were measured for each barley genotype.

For the steady-state ion flux measurements, 3-day-old seedlings were pretreated with 20% PEG8000 in the background of BSM for 1 h, 6 h and 24 h prior to the ion fluxes measurements. Seedlings without PEG treatment were used as the control. Ion fluxes were measured for 10 min, and the steady-state fluxes were calculated by averaging the values of the last 5 minutes. Eight individual roots were measured for each barley genotype.

### 4.8. qRT-PCR

The transcriptional levels of genes for K^+^ transmembrane transport and plasma membrane ATPase were determined in the barley genotypes of Gairdner, Morex and WMLL. Barley seedlings were grown in aerated one-fifth Hoagland solution (pH6.0) until the first leaf was fully expanded (for 7 days) to ensure enough root segments for gene expression analysis. Then, drought stress was induced using polyethylene glycol 8000 (PEG8000, P103734, Aladdin, Shanghai, China) in the background of one-fifth Hoagland solution (pH6.0). Two drought levels were used as control (CK) and 20% PEG8000. The solution was renewed every day. After onset of drought treatment for 1 h, 6 h, 1 d and 6 d, root segments (∼50 mm from the root tip) of barley plants under both control and drought conditions were collected for RNA extraction and qRT-PCR. Three biological replicates were determined for each genotype.

Total RNA was extracted by using MiniBEST Plant RNA Extraction Kit (9769, TaKaRa) following the manufacturer’s instructions. RNA concentrations were determined by NanoDrop2000 spectrophotometer (Thermo Fisher Scientific, Waltham, Massachusetts, USA) and qualities were detected by agarose gel electrophoresis. The cDNA was synthesized from total RNA (1 ug) using PrimeScript RT Reagent Kit (RR037A, TaKaRa, Shiga, Japan) and was used as templates for qPCR amplification. qPCR amplification was performed with LightCycler 480 II (Roche, Basel, Switzerland) using SYBR Green Supermix (Bio-Rad, Berkeley, CA, USA). The primer sequences are listed in the Supplemental Table. Two technical replicates were performed for each biological replicate. The relative gene expression was calculated based on the 2 ^−△△Ct^ method using *Beta-tubulin* as the internal standard [77,78], and compared with the control values at each sampling time point.

### 4.9. Statistical Analysis

Statistical analyses were performed using SPSS Statistics 20 (IBM Corp. Armonk, New York, NY, USA). All data in the figures were given as mean ± SE. Significance of differences was determined by Student’s t-test. One to three asterisks indicate the significance at *p* < 0.05, 0.01 or 0.001level, respectively.

## 5. Conclusions

Our MIFE measurements revealed that drought tolerant barley genotype WMLL exhibited a significantly larger K^+^ influx and H^+^ efflux, compared to the sensitive genotype Gairdner. The current results demonstrated that root K^+^ uptake and root to shoot K^+^ translocation confer the osmotic adjustment and contribute to the genotypic difference in drought tolerance levels in barley. Gene expression measurements showed that the expression of genes conferring K^+^ uptake (*HvHAK1*, *HvHAK5*, *HvKUP1*, *HvKUP2,* and *HvAKT1*) and xylem loading (*HvSKOR*) in roots were all affected by drought stress in a time- and genotype-specific manner, which consequently led to a significant genotypic difference in root and shoot K^+^ contents. These results indicated that the regulation of the K^+^ transporters and channels is critical for root K^+^ uptake and root to shoot K^+^ translocation in barley under drought stress. Furthermore, the barley genotypes showed a strong correlation between H^+^ efflux and K^+^ influx under drought stress, which was further confirmed by the significant up-regulation of *HvHA1* and *HvHA2*, suggesting an important role of PM H^+^-ATPase activity and/or expression in regulating the activity of K transporters and channels under drought stress. Taken together, the ability to regulate potassium transport across plasma membrane in roots is critical for drought tolerance in barley.

## Supplementary Materials

Supplementary materials can be found at https://www.mdpi.com/1422-0067/20/17/4111/s1.

## Abbreviations

AHA	Arabidopsis plasma membrane H^+^-ATPase
AKT1	Arabidopsis K^+^ transporter 1
BSM	Basic salt medium
CBL1	Calcineurin B-like protein 1
CIPK23	CBL-interacting protein kinase 23
DW	Dry weight
FW	Fresh weight
gs	Stomatal conductance
HAK	High-affinity K^+^ transporter
ILK1	Integrin-linked kinase1
KIR	K inward rectifier
KUP	K^+^ uptake transporter
LIX	Liquid ion exchanger
MIFE	Microelectrode ion flux estimation
PEG	Polyethylene glycol
PM	Plasma membrane
PSII	Photosystem II
qRT-PCR	Quantitative reverse transcriptase PCR
RWC	Relative water content
SKOR	Stelar K^+^ outward rectifier
TW	Turgid weight
