# Supplementary material for: The Ability to Regulate Transmembrane Potassium Transport in Root Is Critical for Drought Tolerance in Barley
Source: Int J Mol Sci. 2019 Aug 22;20(17):4111. doi: 10.3390/ijms20174111 (PMC6747136; doi:10.3390/ijms20174111)
Supplement: Supplementary file 1 [file ijms-20-04111-s001.zip › Supplementary Figures.pptx]

## Slide 1
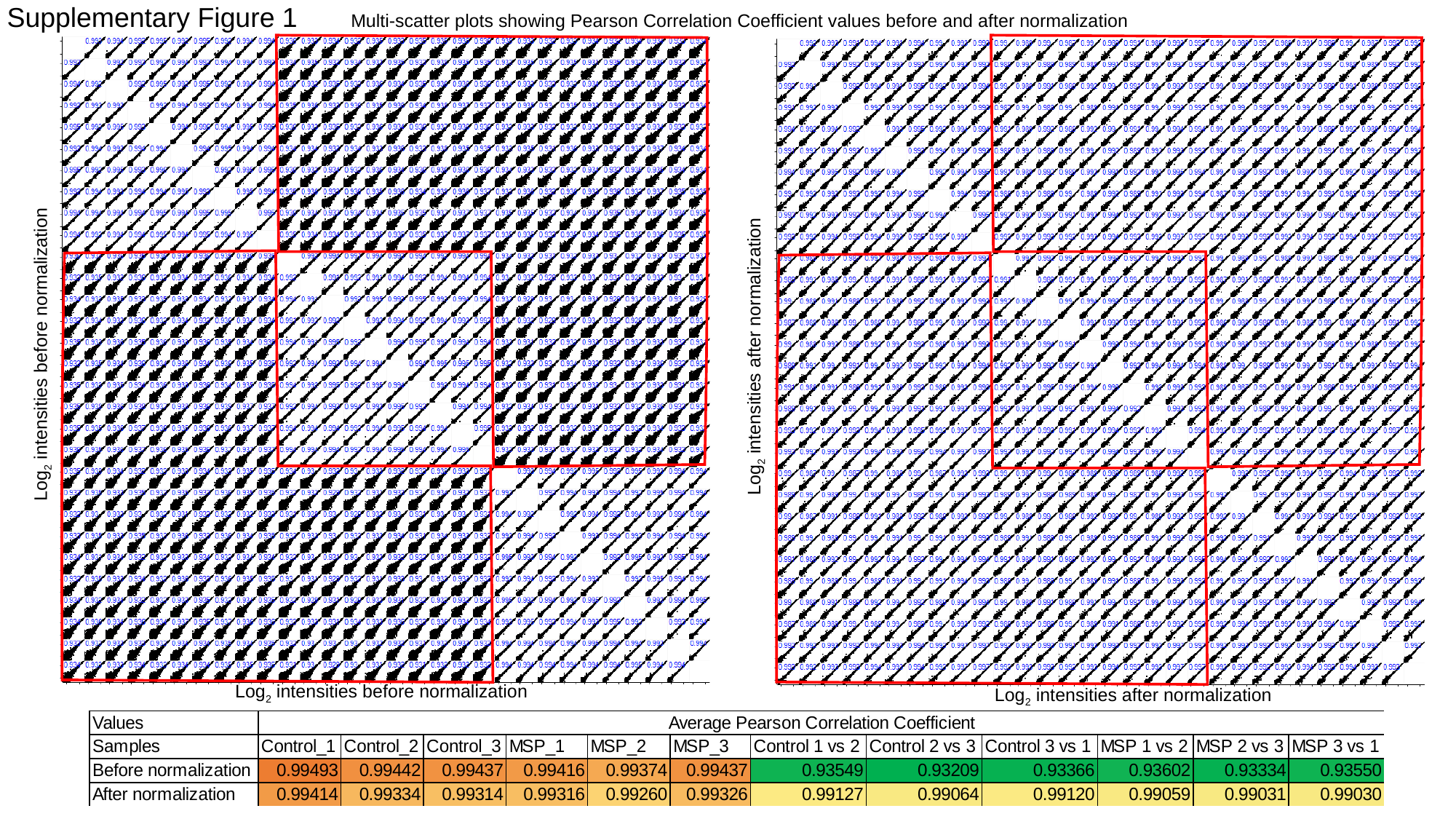

Supplementary Figure 1
Multi-scatter plots showing Pearson Correlation Coefficient values before and after normalization
Log2 intensities before normalization
Log2 intensities before normalization
Log2 intensities after normalization
Log2 intensities after normalization

## Slide 2
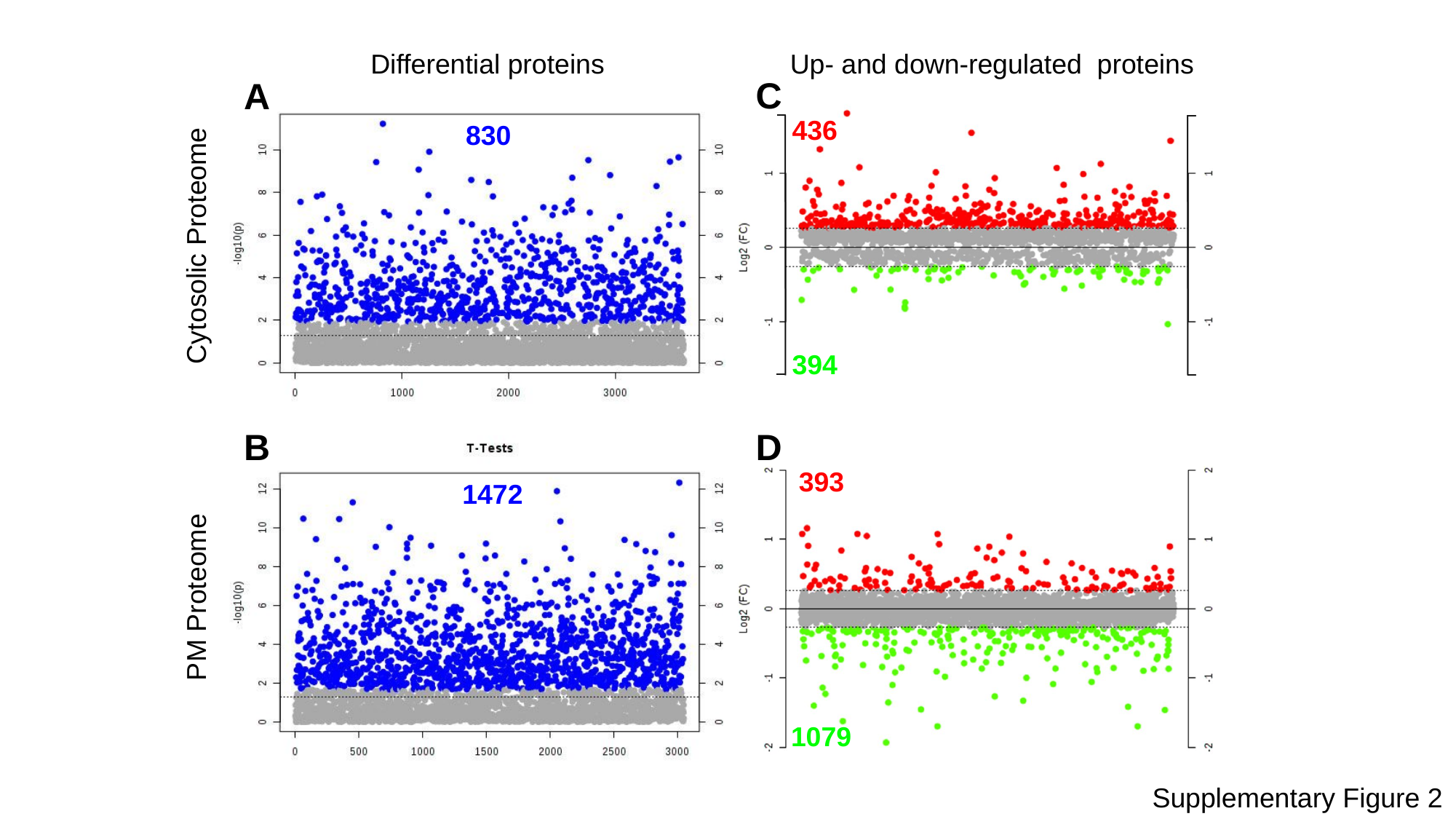

Up- and down-regulated proteins
Differential proteins
C
A
436
394
830
Cytosolic Proteome
D
B
1472
393
1079
PM Proteome
Supplementary Figure 2

## Slide 3
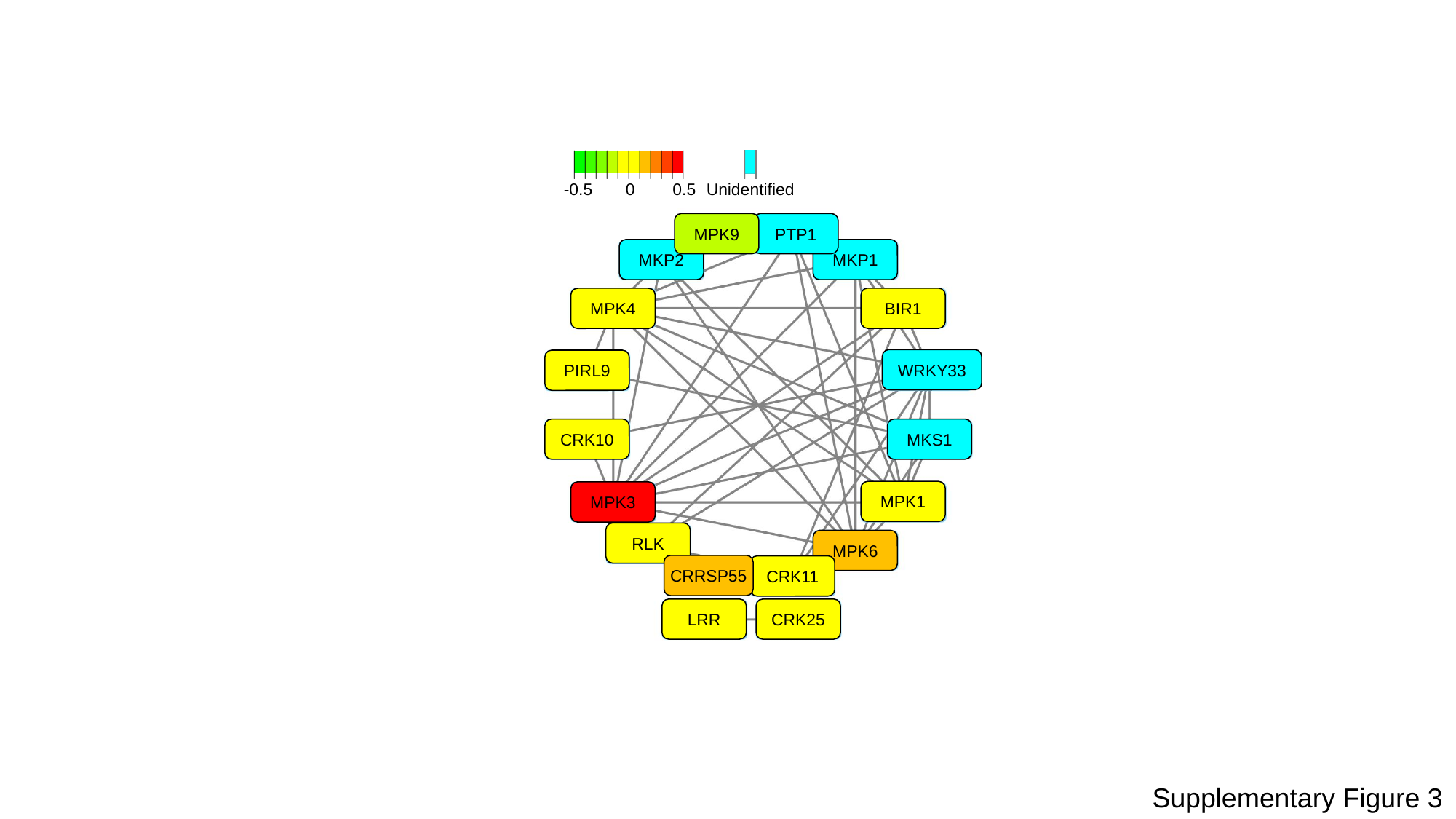

-0.5 0 0.5
Unidentified
MPK9
PTP1
MKP2
MKP1
BIR1
MPK4
WRKY33
PIRL9
CRK10
MKS1
MPK1
MPK3
RLK
MPK6
CRRSP55
CRK11
LRR
CRK25
Supplementary Figure 3

## Slide 4
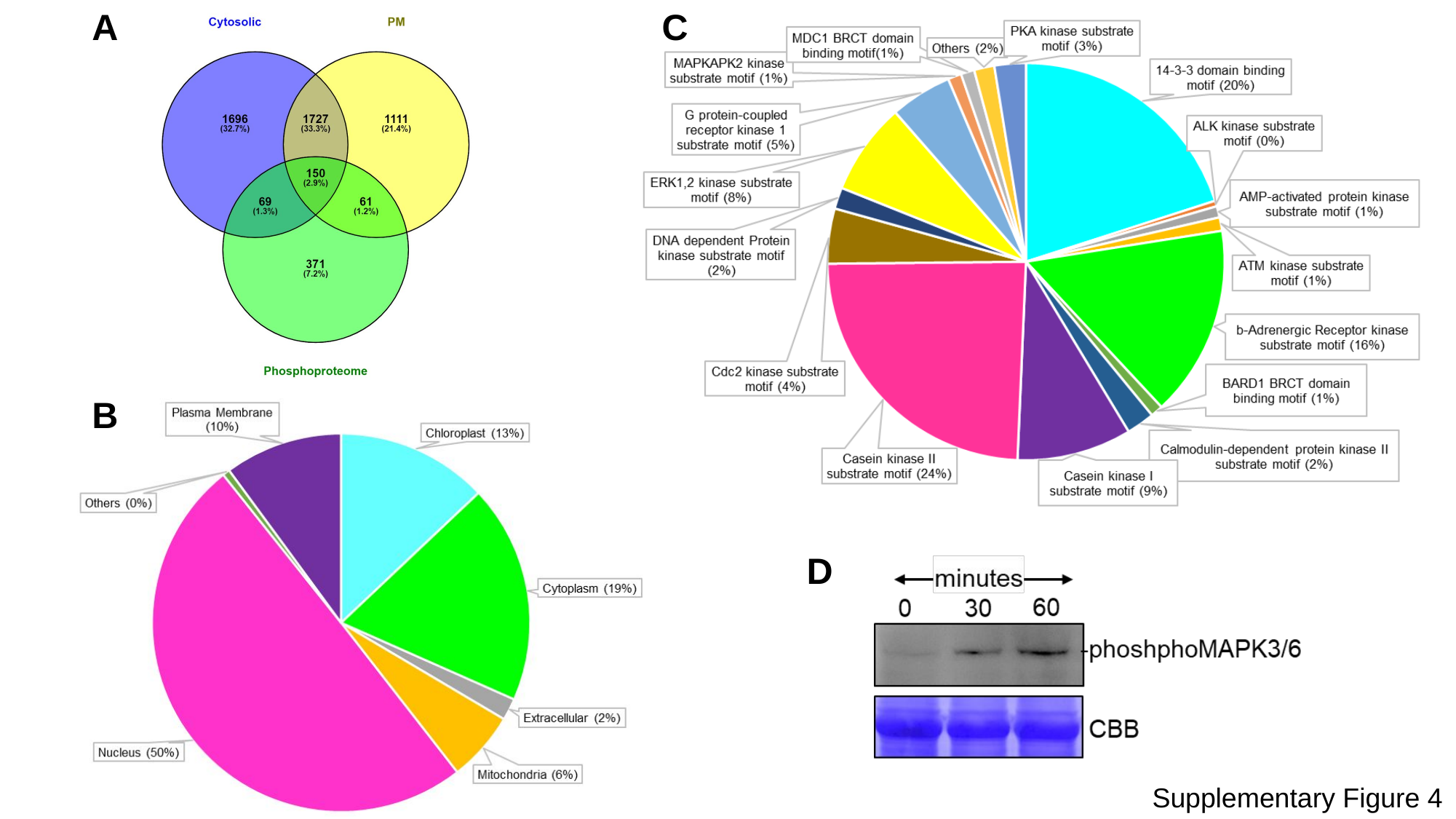

A
C
B
D
Supplementary Figure 4

## Slide 5
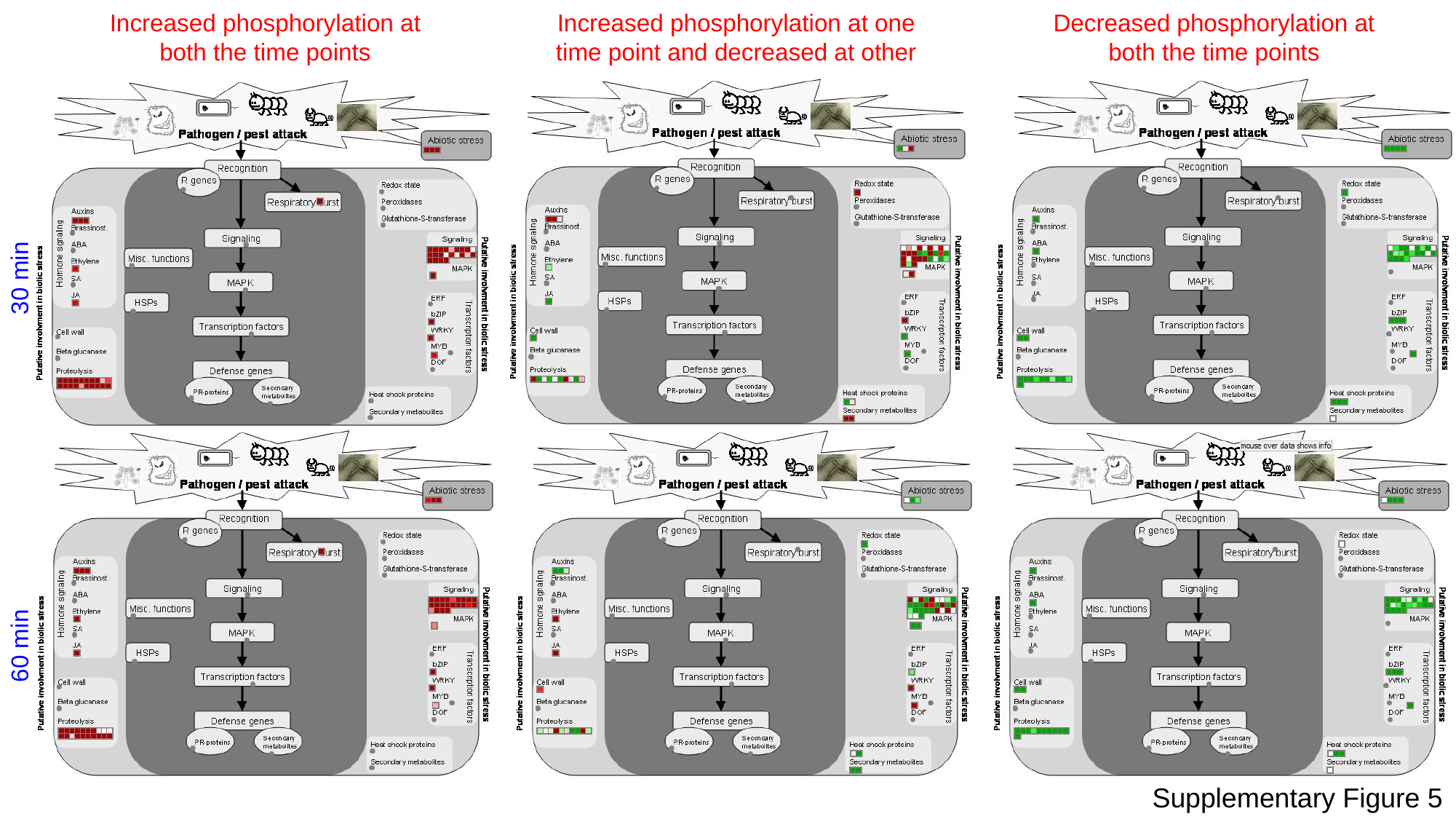

Increased phosphorylation at both the time points
Increased phosphorylation at one time point and decreased at other
Decreased phosphorylation at both the time points
30 min
60 min
Supplementary Figure 5
